# 2015: The Year of Anti-PD-1/PD-L1s Against Melanoma and Beyond

**DOI:** 10.1016/j.ebiom.2015.01.011

**Published:** 2015-01-19

**Authors:** Paolo A. Ascierto, Francesco M. Marincola

**Affiliations:** a Istituto Nazionale Tumori, Fondazione “G. Pascale”, Napoli, Italy; b Sidra Medical and Research Centre, Doha, Qatar

The history of clinical oncology has witnessed several revolutionary therapeutic advances that have significantly improved cancer care. These have included the introduction of cisplatin in the 1970s for testicular and ovarian cancers, the taxanes in the 1990s for breast and other solid tumors, targeted therapy with anti-HER2 for breast cancer and c-Kit inhibitors for chronic myeloid leukemia and other cancers at the start of this millennium. Each of these treatments has revolutionized outcomes for patients with various types of cancer. Today, we are at the start of a new era in cancer care — that of immunotherapy. The approval of sipuleucel-T for the treatment of prostate cancer in 2010 and ipilimumab (anti-CTLA-4) for advanced melanoma in 2011 was the first notable success in the immunotherapy of cancer. After almost three years from the approval of the first checkpoint inhibitor (ipilimumab), the good news is not over. Quite the contrary, we are only at the beginning and, notably, these advances do not relate just to the treatment of melanoma. Immunotherapy has become the fourth pillar of cancer treatment alongside surgery, radiotherapy and chemotherapy (including targeted therapy). This can be attributed primarily to the impact that another group of checkpoint inhibitors, the anti-PD-1/PD-L1 agents, is having on the treatment of various malignancies.

As with anti-CTLA-4, the anti-PD-1/PD-L1 story starts with melanoma. Data from a large phase I study with pembrolizumab (Robert et al., 2014a, Robert et al., 2014b) led to its approval by the US Food and Drug Administration (FDA) in September 2014 for the treatment of patients with unresectable or metastatic melanoma and disease progression following ipilimumab and, if BRAF V600 mutation positive, a BRAF inhibitor. This study of 411 patients showed that pembrolizumab resulted in an overall response rate (ORR) of 34%, a median progression-free survival (PFS) of 5.5 months, and overall survival (OS) rates of 69% at one year and 62% at 18 months. Moreover, a randomized phase II trial with pembrolizumab at two different dosages (2 mg/kg or 10 mg/kg every three weeks) in advanced melanoma refractory to previous ipilimumab therapy showed that both doses improved PFS compared with investigator choice chemotherapy (Ribas et al., 2014). In fact, the 6-month PFS was 34% with pembrolizumab 2 mg/kg, 38% with pembrolizumab 10 mg/kg and 16% with chemotherapy, while the 9-month PFS was 24%, 29% and 8% respectively. The ORR in the three groups was 21%, 25% and 4%, respectively.

More recently, in December 2014, another anti-PD-1, nivolumab, was approved by the FDA for patients with advanced melanoma with the same indication as pembrolizumab. Data from a large phase I trial with nivolumab showed an ORR of 32% and 1, 2, 3, and 4-year OS rates of 63%, 48%, 42%, and 32%, respectively. In addition, data from a phase III study in patients with metastatic melanoma previously treated with ipilimumab reported that nivolumab had an ORR of 32% compared with 11% with the chemotherapy control arm (D'Angelo et al., 2014). Nivolumab was also compared to chemotherapy in another randomized phase III trial in which untreated patients with advanced BRAF wild-type melanoma received either nivolumab or dacarbazine. The ORR was 40.0% in the nivolumab group versus 13.9% in the dacarbazine group. At 1 year, the OS was 73.0% in the nivolumab group compared with 42.1% in the dacarbazine group. Median PFS was 5.1 months in the nivolumab group versus 2.2 months in the dacarbazine group (Robert et al., 2014a, Robert et al., 2014b).

Considering historical data with a typical median OS of 6.2 months and 1-year OS rate of 25.5% and 1 and 2-year OS rates of 45% and 24% obtained with ipilimumab therapy in the treatment of advanced melanoma, the results achieved with anti-PD-1 therapy represent a terrific improvement in clinical benefit for these patients. Moreover, these data obtained in melanoma patients are just the start.

OS data from a phase I study of nivolumab in solid tumors were particularly encouraging, even in patients with non-small-cell lung cancer (NSCLC), with a median OS of 9.6 months, 1-year OS of 42% and 2-year OS of 24%. Moreover, in a phase II study in patients with advanced, refractory NSCLC, nivolumab was associated with an ORR of 15% and a median OS of 8.2 months (Ramalingam et al., 2014). Historically, these patients have ORRs of between 2 and 8% and a median OS of about 5 months. The estimated 1-year survival rate was 41%, which also compares favorably with historical data for patients with third-line squamous cell NSCLC of 1-year OS rates of 5.5–18%. Pembrolizumab has also shown interesting results in NSCLC. In the NSCLC expansion cohort of a phase I trial, pembrolizumab treatment resulted in an ORR of 21%. The median PFS in treatment-naïve patients was 27 weeks and 6-months OS was 86%, while in pretreated patients median PFS was 10 weeks and 6-months OS was 59% (Garon et al., 2014).

Interesting preliminary results have also been reported for urothelial bladder cancer (UBC) and triple negative breast cancer (TNBC). In patients with platinum-pretreated, metastatic UBC, the ORR obtained with an anti-PD-L1 antibody (MPDLA3280) was between 11% and 43%, depending on the level of PD-L1 (Powles et al., 2014). In patients with heavily pretreated advanced TNBC, pembrolizumab achieved an ORR of 18.5% with a durable response (median response duration was not reached) (Nanda et al., 2014).

Anti-PD-1 therapy has also achieved interesting results in patients with hematological malignancies. In a small phase I study that enrolled 23 patients with relapsed or refractory Hodgkin's lymphoma that had already been heavily treated, nivolumab resulted in a clinical benefit in all patients; the ORR was 87% (20/23), with 17% having a complete response and 70% a partial response. The remaining three patients (13%) all had stable disease (Ansell et al., 2014). PFS at 6 months was 86%. In another phase Ib study, pembrolizumab also demonstrated promising antitumor activity in patients with heavily pretreated Hodgkin's lymphoma, with a 21% complete remission rate and an ORR of 65% (Craig et al., 2014).

What can we expect during 2015? New data in other types of cancer are surely expected. Studies with anti-PD-1/PD-L1 are ongoing in gastric cancer, small-cell lung cancer, glioblastoma, colorectal cancer, Merkel cell carcinoma and others. There are also likely to be more data concerning the role of PD-L1 as a predictive marker, even though data from phase III studies in melanoma seem to refute such a role. We will also see more important news on the potential of anti-PD-1/PD-L1s in combination with other approaches, including other immunotherapies (e.g. checkpoint inhibitors), radiotherapy, chemotherapy and targeted agents. We are observing the beginning of another new era in the fight against cancer: that of anti-PD-1/PD-L1 therapy.

## Conflicts of Interest

Paolo A. Ascierto has/had an advisory/consultant role for Bristol Myers Squibb, Roche-Genentech, Merck Sharp & Dohme, Glaxo SmithKline, Ventana, Amgen, and Novartis. He also received research funds from Bristol Myers Squibb, Roche-Genentech, and Ventana. Francesco Marincola has no conflicts of interest to declare.
